# Supplementary material for: Biochemical recovery from exertional heat stroke follows a 16-day time course
Source: PLoS One. 2020 Mar 4;15(3):e0229616. doi: 10.1371/journal.pone.0229616 (PMC7055888; doi:10.1371/journal.pone.0229616)
Supplement: S1 Table — (PDF) [file pone.0229616.s001.pdf]

S1 Table. Clinical diagnostics, laboratory tests associated with heat stroke diagnosis and medical evaluation

Primary clinical evaluation, physiologic parameters

Analyte	Organ system, Function
Core body temperature (esophageal or rectal)	Thermoregulatory system
Heart rate	Cardiovascular
Blood pressure (systolic/diastolic)	Cardiovascular

Laboratory evaluation (Blood, Urine, Serum) *Multi-organ injury / dysfunction measures*

Laboratory Panel	Analyte	Organ system, Function
**Metabolic Panel** (may also see *Electrolyte* or *Renal Panel*)	Sodium, Potassium, Chloride, Bicarbonate, Calcium, Blood Urea Nitrogen, Creatinine, Glucose (blood), Glomerular filtration rate	Kidney, Metabolism
**Liver Function Test** (LFT)	Alanine Aminotransferase, Aspartate Aminotransferase, Alkaline Phosphatase, Albumin, Total protein, Bilirubin, Lactate Dehydrogenase, Prothrombin Time	Liver, Coagulation
**Other Circulating Enzymes / Electrolytes** (Myoloysis, Hepatolysis)	Creatine Phosphokinase, Myoglobin (serum), Magnesium, Phosphorus, Uric acid, Lactic acid	Muscle (myolysis), Metabolism
**Complete Blood Count** (CBC)	Red blood cell count, Hemoglobin, Hematocrit, White blood cell count, Platelet count (may also include differential: Neutrophils, Eosinophils, Basophils, Leukocyte, Lymphocyte)	Hematology, Immune function
**Coagulation**	Prothrombin time, Partial thromboplastin time, International normalized ratio	Coagulation, Liver
**Urinalysis** (UA)	Specific gravity, Glucose (urine), Myoglobin (urine), Bilirubin (urine), Urobilinogen, Blood (urine), Protein (urine), Nitrites, pH, Color, Clarity, Nitrites, Ketone, Leukocyte esterase test	Kidney, Metabolism, Liver

Ancillary clinical evaluation

Analysis	Analyte	Organ system, Function
Chest x-ray	Infiltrate'	Pulmonary (infection)
Electrocardiogram	Full (12-lead) EKG	Cardiovascular
Arterial blood gas	PaO_2, $PaCO_2$, pH, HCO_3^-, O_2CT, O_2Sat	Pulmonary, Cardiovascular
Toxicological / Drug Screening	*Specifically cocaine, alcohol	*Intoxication*

Adapted from:

US Army. Technical Bulletin Medicine 507:"Heat Stress and Heat Casualty Management" (TBMED 507), 2003.

US Navy. Technical Manual 6260.6A "Prevention and Treatment of Heat and Cold Injuries" (NEHC-TM-OEM 626), June 2007.
