# Supplementary material for: Biochemical recovery from exertional heat stroke follows a 16-day time course
Source: PLoS One. 2020 Mar 4;15(3):e0229616. doi: 10.1371/journal.pone.0229616 (PMC7055888; doi:10.1371/journal.pone.0229616)
Supplement: S2 Table — (PDF) [file pone.0229616.s002.pdf]

S2 Table. Serum Electrolytes/Metabolites – Mean Laboratory Values and Percent of Patient Relative to Reference Range, 0-2 days post EHS

VARIABLE (REF RANGE)		DAY 0		DAY 1		DAY 2
**Sodium** (136-145 mmol/L)	MEAN ± SD	140.2 ± 3.8		139.9 ± 2.5		140.1 ± 2.5
	PTs ABOVE REF	**7%**	(+)	1%		1%
	PTs BELOW REF	**9%**	(-)	3%		4%
**Urea Nitrogen** (7-18 mg/dL)	MEAN ± SD	17.3 ± 5.9		13.9 ± 5.3		10.1 ± 4.2
	PTs ABOVE REF	**34%**	(+)	17%		3%
	PTs BELOW REF	0%		3%		17%
**Glucose** (74-106 g/dL)	MEAN ± SD	102.1 ± 34.9		92.5 ± 13.1		95 ± 20.1
	PTs ABOVE REF	**30%**	(+)	12%		14%
	PTs BELOW REF	**12%**	(-)	3%		5%
**Potassium** (3.4-5.0 mmol/L)	MEAN ± SD	4.2 ± 0.5		3.9 ± 0.4		4.1 ± 0.4
	PTs ABOVE REF	**5%**	(+)	0%		1%
	PTs BELOW REF	**4%**	(-)	5%		1%
**Calcium** (8.6-10.6 g/dL)	MEAN ± SD	9.3 ± 0.8		**8.3** ± 0.6	(L)	8.7 ± 0.6
	PTs ABOVE REF	6%		0%		0%
	PTs BELOW REF	18%		**68%**	(-)	44%
**Carbon Dioxide** (21-32 mmol/L)	MEAN ± SD	22.8 ± 4.5		24.4 ± 3.0		26.3 ± 3.1
	PTs ABOVE REF	0%		1%		2%
	PTs BELOW REF	**27%**	(-)	10%		5%
**Chloride** (98-107 mmol/L)	MEAN ± SD	103.5 ± 4.9		**107.4** ± 3.6	(H)	106.4 ± 3.5
	PTs ABOVE REF	20%		**54%**	(+)	40%
	PTs BELOW REF	9%		1%		1%
**Bilirubin (total),** (0.2-1.0 mg/dL)	MEAN ± SD	0.85 ± 0.56		0.78 ± 0.56		0.72 ± 0.65
	PTs ABOVE REF	**25%**	(+)	19%		16%
	PTs BELOW REF	0%		1%		3%
**Creatinine,** (0.66-1.25 mg/dL)	MEAN ± SD	**1.4** ± 0.5	(H)	1.1 ± 0.4		1.0 ± 0.4
	PTs ABOVE REF	**58%**	(+)	17%		9%
	PTs BELOW REF	1%		2%		2%

(H) Mean value exceeds upper limit of reference range
(L) Mean value exceeds lower limit of reference range
(+) Patient population peak with 'hyper'-emic state
(-) Patient population peak with 'hypo'-emic state
