# Supplementary material for: Biochemical recovery from exertional heat stroke follows a 16-day time course
Source: PLoS One. 2020 Mar 4;15(3):e0229616. doi: 10.1371/journal.pone.0229616 (PMC7055888; doi:10.1371/journal.pone.0229616)
Supplement: S3 Table — (PDF) [file pone.0229616.s003.pdf]

S3 Table. Abnormal Urinalysis Findings in Patients, during 14 days of EHS Follow-Up

**VARIABLE** (REF RANGE)		**DAY 0**	**DAY 1**	**DAY 2**	**DAY 3**	**DAY 4**	**DAY 5**	**DAY 6**	**DAY 7**	**DAY 10**	**DAY 14**
**Protein**[1] (Negative)	% ABNORMAL	72%	40%	10%	14%	15%	23%	10%	5%	7%	10%
**Blood**[2] (Negative)	% ABNORMAL	45%	40%	34%	32%	27%	18%	13%	17%	10%	10%
**Ketones**[3] (Negative)	% ABNORMAL	46%	37%	37%	12%	7%	4%	2%	0%	3%	3%
**Myoglobin**[4] (Negative)	% ABNORMAL	17%	10%	9%	5%	8%	14%	25%	11%	0%	0%
**Glucose**[5] (Negative)	% ABNORMAL	15%	4%	2%	3%	1%	0%	0%	0%	0%	0%
**Color**[6] (Yellow)	% ABNORMAL	5%	2%	1%	1%	2%	3%	2%	0%	0%	0%
**Bilirubin**[7] (Negative)	% ABNORMAL	2%	1%	0%	2%	3%	0%	0%	0%	0%	0%
**Nitrites**[8] (Negative)	% ABNORMAL	<1%	<1%	<1%	0%	0%	0%	0%	0%	0%	0%

1 Protein - Abnormal if reported as "Large, Moderate, Small, Trace, 3+, 2+, 1+, or value '>0"
2 Blood - Abnormal if reported as "Large, Moderate, Small, Trace (lysed or intact), 3+, 2+, 1+, or any value '>0"
3 Ketones - Abnromal if reported as "Large, Moderate, Small, Trace, 4+, 3+, 2+, 1+, or any value '>0"
4 Myoglobin - Abnormal if reported as "Positive" or 'any value >0'
5 Glucose - Abnormal if reported as "Moderate, Small, Trace, 3+, 2+, 1+, or value '>0"
6 Color - Abnormal if reported as "Amber, Orange, Dark Orange, Light Red, Brownish Red, Red, Port Wine, Brown"
7 Bilirubin - Abnormal if reported "Moderate, Small, Trace or value '>0"
8 Nitrites - Abnormal if reported as "Positive"
