# Supplementary material for: Biochemical recovery from exertional heat stroke follows a 16-day time course
Source: PLoS One. 2020 Mar 4;15(3):e0229616. doi: 10.1371/journal.pone.0229616 (PMC7055888; doi:10.1371/journal.pone.0229616)
Supplement: S4 Table — (PDF) [file pone.0229616.s004.pdf]

**S4 Table. Liver Function/Muscle Injury - Median Laboratory Values in Patients, during 14 days of EHS Follow-Up**

| **VARIABLE** (Ref Range) | | **DAY 0** | **DAY 1** | **DAY 2** | **DAY 3** | **DAY 4** | **DAY 5** | **DAY 6** | **DAY 7** | **DAY 10** | **DAY 14** |
|---|---|---|---|---|---|---|---|---|---|---|---|
| **Alanine Aminotransferase** | MEDIAN | 35 | 68 (H) | 143 (H) | **155.5** **(H)*** | **155.5** **(H)*** | 151 (H) | 125.8 (H) | 122 (H) | 112 (H) | 57 (H) |
| (6-43IU/L) | IQR | (24 - 52) | (38 - 145) | (65 - 389.7) | (76.1 - 420.3) | (76.5 - 457.5) | (62 - 385) | (58 - 310.8) | (56 - 278.5) | (43 - 208.5) | (30 - 105) |
| | PTs ABOVE REF | 36% | 70% | 87% | 89% | 85% | 87% | 85% | 81% | 75% | 62% |
| | PTs BELOW REF | 0% | 0% | 0% | 0% | 0% | 0% | 0% | 0% | 0% | 0% |
| **Aspartate Aminotransferase** | MEDIAN | 43 (H) | 95 (H) | **176.2** **(H)*** | 126.5 (H) | 96 (H) | 78 (H) | 56 (H) | 51 (H) | 38.5 | 31.5 |
| (10-40 IU/L) | IQR | (29 - 70.7) | (49.5 - 232.5) | (73.9 - 411) | (58.4 - 289.3) | (48 - 290.5) | (43.5 - 204) | (31 - 137.5) | (30.5 - 105.5) | (26 - 61.8) | (24 - 47.8) |
| | PTs ABOVE REF | 55% | 83% | 87% | 86% | 78% | 78% | 63% | 59% | 49% | 33% |
| | PTs BELOW REF | 0% | 0% | 0% | 0% | 0% | 0% | 0% | 0% | 0% | 0% |
| **Creatine Kinase** | MEDIAN | 503 (H) | 1138 (H) | 1236 (H) | **1251** **(H)*** | 700 (H) | 708 (H) | 484 (H) | 276 (H) | 222 | 153.5 |
| (22-269 IU/L) | IQR | (238 - 1202) | (521 - 3744) | (460 - 5295) | (321 - 5257) | (242 - 3374) | (237 - 3100) | (172 - 1164) | (152 - 907) | (99 - 552) | (103 - 366) |
| | PTs ABOVE REF | 71% | 90% | 85% | 81% | 72% | 70% | 62% | 51% | 44% | 37% |
| | PTs BELOW REF | 0% | 0% | 0% | 0% | 0% | 0% | 0% | 0% | 0% | 0% |
| **Myoglobin (Serum)** | MEDIAN | **145** **(H)*** | 120 (H) | 71 | 82 (H) | 78 (H) | 72 (H) | 66 | 40 | 35 | 41 |
| (28-72 µg/L) | IQR | (38 - 523) | (70 - 345) | (41 - 264) | (35 - 160) | (42 - 214) | (43 - 141) | (42 - 118) | (34 - 70) | (34 - 51) | (28 - 57) |
| | PTs ABOVE REF | 55% | 70% | 50% | 51% | 48% | 45% | 36% | 20% | 0% | 13% |
| | PTs BELOW REF | 10% | 2% | 9% | 8% | 0% | 10% | 3% | 14% | 15% | 17% |
| **Alkaline Phosphatase** | MEDIAN | 72 | 60 | 62 | 66 | 69 | 67 | 69.5 | 73 | 69 | 69 |
| (38-126 IU/L) | IQR | (59.6 - 87) | (50 - 72.4) | (50.5 - 77.5) | (54.5 - 80) | (58 - 81) | (58 - 78) | (60 - 87) | (58 - 85) | (60 - 84) | (61 - 84) |
| | PTs ABOVE REF | 3% | 2% | 2% | 2% | 4% | 4% | 9% | 3% | 5% | 0% |
| | PTs BELOW REF | 1% | 5% | 4% | 3% | 0% | 2% | 0% | 0% | 3% | 0% |
| **Albumin** | MEDIAN | 4.4 | 3.5 | 3.5 | 3.7 | 4.1 | 4.3 | 4.4 | 4.5 | 4.4 | 4.6 |
| (3.4-5.0 IU/L) | IQR | (4 - 4.8) | (3.2 - 3.8) | (3.2 - 3.9) | (3.3 - 4.3) | (3.4 - 4.5) | (3.7 - 4.7) | (4.1 - 4.7) | (4.1 - 4.7) | (4.1 - 4.7) | (4.3 - 4.8) |
| | PTs ABOVE REF | 12% | 1% | 0% | 1% | 2% | 4% | 6% | 3% | 3% | 5% |
| | PTs BELOW REF | 3% | 38% | 37% | 30% | 24% | 19% | 15% | 12% | 8% | 0% |
| **Lactate Dehydrogenase** | MEDIAN | 446 | 454 | 475 | 389 | 444 | 368 | 276 | 292 | 256 | 230 |
| (313-618 IU/L) | IQR | (266 - 715) | (255 - 718) | (264 - 861) | (271 - 756) | (248 - 826) | (270 - 564) | (226 - 557) | (189 - 501) | (227 - 348) | (182 - 163) |
| | PTs ABOVE REF | 35% | 33% | 36% | 33% | 38% | 22% | 20% | 14% | 15% | 13% |
| | PTs BELOW REF | 34% | 34% | 34% | 38% | 34% | 39% | 57% | 50% | 70% | 63% |

*(H) Value exceeds upper limit of reference range*
***(H)**** *Peak value, exceeds upper limit of reference range*
