# Supplementary material for: Biochemical recovery from exertional heat stroke follows a 16-day time course
Source: PLoS One. 2020 Mar 4;15(3):e0229616. doi: 10.1371/journal.pone.0229616 (PMC7055888; doi:10.1371/journal.pone.0229616)
Supplement: S5 Table — (PDF) [file pone.0229616.s005.pdf]

S5 Table. Blood Counts & Coagulation - Mean Laboratory Values and Percent of Patient-Values Relative to Reference Range, during 14 days of EHS Follow-Up

VARIABLE (REF RANGE)		DAY 0		DAY 1		DAY 2		DAY 3		DAY 4		DAY 5		DAY 6		DAY 7		DAY 10		DAY 14	
**Hematocrit**	MEAN ± SD	42.3 ± 4.3		**37.7** ± 3.4	**(L)***	38.2 ± 3.6	(L)	38.9 ± 4.0	(L)	40.0 ± 5.0	(L)	40.5 ± 5.7	(L)	41.6 ± 5.8	(L)	41.7 ± 5.6	(L)	41 ± 5.8	(L)	43 ± 5.1	
(42-52%)	PTs ABOVE REF	1%		0%		0%		0%		0%		1%		0%		0%		2%		0%	
	PTs BELOW REF	47%		**90%**	(-)	85%		76%		62%		52%		43%		41%		53%		27%	
**Hemoglobin**	MEAN ± SD	14.4 ± 1.53		**12.83** ± 1.2	**(L)***	**12.9** ± 1.3	(L)	**13.2** ± 1.4	(L)	**13.6** ± 1.7	(L)	**13.8** ± 1.9	(L)	14.1 ± 2		14.1 ± 1.9		**13.9** ± 1.95	(L)	14.5 ± 1.8	
(14.0-18.0 g/dL)	PTs ABOVE REF	1%		0%		0%		0%		0%		0%		0%		0%		0%		0%	
	PTs BELOW REF	38%		86%	**(+)**	79%		71%		55%		46%		35%		35%		44%		24%	
**Red Blood Cells**	MEAN ± SD	4.8 ± 0.5		**4.2** ± 0.4	**(L)***	4.3 ± 0.4	(L)	4.4 ± 0.5	(L)	4.5 ± 0.6	(L)	4.5 ± 0.6	(L)	4.6 ± 0.7	(L)	4.6 ± 0.6	(L)	4.6 ± 0.6	(L)	4.9 ± 0.6	
(4.7-6.1 x 10^6 cells/µL)	PTs ABOVE REF	1%		0%		0%		0%		1%		0%		1%		0%		0%		3%	
	PTs BELOW REF	47%		**87%**	(-)	84%		75%		60%		59%		43%		42%		49%		24%	
**White Blood Cells**	MEAN ± SD	**11.6** ± 4.9	**(H)***	8.7 ± 3.1		7.5 ± 2.6		7.4 ± 2.5		7.5 ± 2.6		7.2 ± 2.9		7.7 ± 2.7		7.8 ± 3.4		6.8 ± 2.8		7.2 ± 3	
(4.9-10.1 x 10^3 cells/µL)	PTs ABOVE REF	**57%**	(+)	26%		13%		11%		10%		9%		15%		14%		9%		8%	
	PTs BELOW REF	3%		4%		7%		11%		8%		10%		9%		9%		18%		11%	
**Neutrophils**	MEAN ± SD	**8.4** ± 4.8	**(H)***	6.0 ± 3.1		5.1 ± 3.0		5.2 ± 3.3		6.2 ± 5.1		4.6 ± 2.0		5.0 ± 2.2		5.1 ± 2.6		4.0 ± 2.2		4.6 ± 2.7	
(1.4-6.5 x 103 cells/µL)	PTs ABOVE REF	**55%**	**(+)**	33%		19%		19%		24%		13%		20%		19%		10%		21%	
	PTs BELOW REF	0%		0%		0%		0%		0%		0%		1%		1%		8%		3%	
**Platelets**	MEAN ± SD	237 ± 67		178 ± 54		173 ± 57		181 ± 66		201 ± 69		216 ± 76		233 ± 79		251 ± 69		348 ± 120		282 ± 107	
(150-450 x 10^3 cells/µL)	PTs ABOVE REF	1%		0%		0%		0%		1%		2%		1%		1%		18%		11%	
	PTs BELOW REF	6%		31%		**35%**	(-)	31%		22%		14%		14%		6%		0%		0%	
**Prothromin Time**	MEAN ± SD	13.6 ± 2.3	(H)	16.4 ± 7.4	(H)	19.3 ± 16.4	(H)	18 ± 17.6	(H)	**21.7** ± 22.9	**(H)***	20.4 ± 18.7	(H)	15.3 ± 4.7	(H)	13.4 ± 3.4	(H)	14.4 ± 2.5	(H)	14 ± 1.3	(H)
(9.5-11.5 secs)	PTs ABOVE REF	83%		**88%**	(+)	83%		70%		71%		65%		78%		58%		81%		100%	
	PTs BELOW REF	0%		0%		0%		0%		0%		0%		0%		0%		0%		0%	

(H) Value exceeds upper limit of reference range
(L) Value exceeds lower limit of reference range
(+) Patient population peak with 'hyper'-emic state
(-) Patient population peak with 'hypo'-emic state

(H)* Peak value, exceeds upper limit of reference range
(L)* Nadir value , exceeds lower limit of reference range
