# Supplementary material for: Biochemical recovery from exertional heat stroke follows a 16-day time course
Source: PLoS One. 2020 Mar 4;15(3):e0229616. doi: 10.1371/journal.pone.0229616 (PMC7055888; doi:10.1371/journal.pone.0229616)
Supplement: Attachment — Submitted filename: 1_Response to Reviewers.docx [file pone.0229616.s006.docx]

To: Christopher James Tyler, PhD, Academic Editor, PLOS ONE

From: Dr Lisa Leon

Subject: Response to Reviewers Comments, MS ID# PONE-D-19-27217
TITLE: Biochemical recovery from exertional heat stroke follows a 16-day time course

Dear Dr. Tyler,

On behalf of the co-authors and myself, thank you for your time, consideration and for providing the reviewers’ comments. The reviews were just and reasonable, and we have made our best effort to satisfy their concerns (see Response to Reviewer Comments, below).

As for formatting, me made significant changes to the manuscript to abide by PLOS ONE requirements, to include adjustments to the style template (editor’s comment #1), separating figure files and supplemental information files (editor’s comment #2), and embedding the tables into the manuscript (editor’s comment #6). We also added information in the methods section as to how laboratory measurements were made (editor’s comment #3 – see line 91-93):

*Laboratory measurements were performed at each treatment location using equipment and procedures as indicated by local medical practice.*

We also amended the ethics statement (editor’s comment #4, see line 93-96):

*The study was reviewed by the United States Army Research Institute of Environmental Medicine and the United States Army Medical Research and Materiel Command institutional review boards and determined this research exempt from written informed consent requirements.*

The amended financial disclosure is included below:

*This work was supported by the United State Department of Defense, Defense Health Program, grant number W81XWH-13-MOMJPC5-IPPEHA (authored by LRL; https://cdmrp.army.mil/dmrdp/default). The funders had no role in study design, data collection and analysis, decision to publish, or preparation of the manuscript.*

Please let us know if any addition modifications are required, or if we can provide any clarification. We look forward to hearing your final determination.

Thank you again.

Very respectfully,

Dr. Lisa Leon

***

Response to the editor, reviewer’s comments:

We thank the reviewer for their careful consideration of our manuscript and helpful comments to assist with our revision. We have responded to each of the comments below and indicate where the text was amended in the manuscript.

[Comments to Author #3]

3. Have the authors made all data underlying the findings in their manuscript fully available?

The PLOS Data policy (<http://www.plosone.org/static/policies.action#sharing>) requires authors to make all data underlying the findings described in their manuscript fully available without restriction, with rare exception (please refer to the Data Availability Statement in the manuscript PDF file). The data should be provided as part of the manuscript or its supporting information, or deposited to a public repository. For example, in addition to summary statistics, the data points behind means, medians and variance measures should be available. If there are restrictions on publicly sharing data—e.g. participant privacy or use of data from a third party—those must be specified.

Reviewer #1: Yes
Reviewer #2: No

Response to [Comments to Author #3]

Thank you for making us aware of this requirement. In accordance with PLOS ‘Sharing Materials’ policy, the authors will make all relevant materials available as part of the manuscript and is submitted along with our revision.

5. Review Comments to the Author

Reviewer #1: The purpose of this manuscript is to document the time course recovery of clinical biomarkers following a heat stroke event in the US Armed Forces. This paper adds to the body of knowledge and will be helpful in developing a targeted and data-driven approach to heat stroke recovery and return to activity/duty/work. There are a couple of items that warrant further clarification within the manuscript.

1) Within the introduction (L26-28) the authors discuss a consensus on EHS RTP/RTD. It may be helpful to provide additional sources of consensus as it relates to sport (NATA Position Statement on Exertional Heat Illness, 2015) and occupational (if any are available) on EHS recovery.

Response to Reviewer Comment #1-1

We value the reviewer’s suggestion and have added the following text below:

Amended [Line 31-35]

“Previous studies have failed to build a consensus or generalizable guideline for normalization of clinical laboratory markers following EHS, even though these markers are currently used as the primary indicator of EHS recovery *by the American College of Sport Medicine (ACSM), the National Athletic Trainers’ Association (NATA), and the United States Army (1,20–22)*.”

2) L75: The authors mention pre-determined patient demographic and clinical variables within the methodology. It would be helpful to include an explanation of what these variables were at this point in the manuscript.

Response to Reviewer Comment #1-2

The text has been modified to list variables included in the this analysis:

Added [Line 77-83]

“Only pre-determined variables were drawn from de-identified EHRs in accordance with the minimum necessary standards of US federal law for patient privacy protections. This analysis included patient demographic variables including age, gender, body mass index, and race, and variables associated with patient care including clinical laboratory values, follow-up care information, and total treatment costs. Total treatment cost were calculated as the cumulative cost to the health care system including initial care and all follow-up or in-patient care associated with the initial injury.”

3) Can the authors provide a rationale for their selection of 60 days here?

Response to Reviewer Comment #1-3

We apologize for not providing a rationale in the original manuscript, as we did put considerable thought into this to determine the best range. The rationale has been provided with the text amended to read:

Added [Line 94-99]

“The 60-day washout period to delineate episodes was based on personal communication with a uniformed physician familiar with EHS follow-up care in the military system, as well as broad risk-based considerations for planning appropriate and timely medical follow-up (29).”

4) Within the discussion (L183 - 206), there is a discussion on the interpretation of clinical values and implications for treatment and return to activity, however, more discussion is needed surrounding the implications for return to activity here. The authors reiterate their findings from a liver damage/dysfunction (L188-191) and urinalysis (L202-206), however, there is no discussion on how return to activity should be guided in these instances. Should all activity cease until these values are within normal limits? Would activity in a cool environment (as recommended based on current consensus) be appropriate? This is an important section of the discussion and should be used to provide some insight on the current clinical recommendations on return to activity and how those may (or may not align) with the findings from this study.

Response to Reviewer Comment #1-4

We greatly appreciate the reviewer’s comments. We have added discussion as to how return to activity should be guided by clinical laboratory values (and physician’s evaluation), and how our findings align with ACSM/NATA/US Army recommendations.

Added [Lines 254-262]

“Consensus recommendations for return to activity by the NATA, ACSM and US Army identify either 7 or 14 days as the minimum post-incident rest period before starting a gradual return to activity or training. Each of these consensus recommendations also indicate ‘normal blood-work results’ and/or medical clearance from a physician’s evaluation as a more individualized indicator of medical readiness. The duration of biochemical recovery described in this analysis strongly supports a minimum 14-day recovery period. Based on the observed variability in biochemical recovery patterns among individuals, a 7-day physical examination including laboratory analysis would enable an earlier return to activity for less severe presentation of EHS.”

As for the other queries listed below, we were very cautious when crafting the discussion for the points mentioned. We feel that these discussion topics extend beyond the mentionable scope of the study and, if included, would be highly speculative in nature. There is insufficient data from this particular study to support these discussion topics. An experimental study or prospective clinical study would better address these questions in order to determine correlation or causation with respect to the appearance of biomarkers in the blood and the incidence of a recurring event..

<<2) Should all activity cease until these values are within normal limits? >>

<<3) Would activity in a cool environment (as recommended based on current consensus) be appropriate? >>

From NATA guidelines (ref 20):

“In all cases of EHS, after the patient has completed a 7- to 21-day rest period, demonstrated normal blood-work results, and obtained physician clearance, he or she may begin a progression of physical activity.”

From ACSM guidelines (ref 1):

“Refrain from exercise for at least 7 d following release from medical care; Follow up about 1 wk postincident for physical examination, and lab testing or diagnostic imaging of affected organs based on the clinical course of the heat stroke incident”

5) In table 2, please indicate that the amplitude of the peak is in reference to the relative fold change compared to ULL/LLN

Response to Reviewer Comment #1-5

Table 2 has be adjusted for clarification, and now indicates each variable as either ‘above ULN’ or ‘below LLN’.

Reviewer #2: I read this article with great interest as it serves to illuminate an area that remains poorly defined in the literature. The manuscript discusses 2,529 exertional heat stroke (EHS) events, and the observed biochemical analysis during recovery. The authors additionally note thatthese labs are critical in the prognosis and return to duty decisions. The authors then subsequently describe a profile of laboratory recovery concluding that the biochemical recovery spans sixteen days. This summary, while very useful in describing a large cohort, however, is somewhat misleading and fails to address previously described and well documented variation dependent upon clinical presentation and management, in the section on limitations.

The value of laboratory tests and the biochemical profile is first and foremost critical in initial management decisions for clinicians. The tone of the manuscript appears to ignore the critical role of management in the impact on the biochemical profile, almost to the point of suggesting that management differences may have little impact on the shape of the biochemical profiles's recovery. The literature is robust with articles on EHS demonstrating the key impact of time to cooling on EHS parameters at presentation, to include morbidity and mortality and the need for dialysis. The roles of aggressive hydration, intra-vascular cooling in management also have shown an impact on the biochemical profile, and clinical recovery. Accordingly, while clearly this is a value added contribution to the literature in a global description of profiles in non fatal EHS events, I strongly believe the data could be interpreted as misleading in failing to recognize the uniqueness of each EHS event with the critical role of early cooling and its impact, as well as the role of effective management. I would argue that time to cooling and management have an impact on the biochemical profile.

Several article to consider:

Varghese GM. Predictors of multi-organ dysfunction in heatstroke.

Emerg Med J. 2005;22:185-187.

Mil Med. 2009 May;174(5):496-502.

Exertional heatstroke: early recognition and outcome with aggressive combined cooling--a 12-yearexperience.

Sithinamsuwan P1

A Tale of Two Heat Strokes: A Comparative Case Study. Stearns RL, Casa DJ, O'Connor FG, Lopez RM. Curr Sports Med Rep. 2016 Mar-Apr;15(2):94-7

These articles and others emphasize the most important point in that all EHS events are not the same, and management strategies can be dramatically different depending upon initial risk stratification, which will impact biochemical profiles. This concept has to be clearly recognized as a study limitation, and reality of EHS management.

Response to Reviewer Paragraph #2-1

We greatly appreciate the reviewer’s critical evaluation of the manuscript. However, we believe this reviewer has generally misinterpreted our primary findings, and is asking us to provide conjecture that goes beyond and fails to acknowledge the study’s principal objectives. To be specific, our focus was to (i) create a generalizable biochemical profile using a large multi-institution cohort, and (ii) characterize the longitudinal nature of biochemical recovery, using data spanning 0-340 days after the initial injury.

The reviewer has instead focused on injury presentation, treatment variables, and the role of the initial biochemical signature on early patient management and medical decision-making. The reviewer focuses on only those variables that would be recorded within the first 1-3hrs after arrival in an emergency care setting.

Furthermore, treatment modalities (intravascular cooling, aggressive hydration, and time-to-cooling/immediacy of care) are largely inaccessible in the electronic health record, and beyond the scope of this study for several reasons. First, EHS treatment often occurs in a ‘field’-type environment, or in an emergency care setting. Treatment information is disparately and non-uniformly annotated in the electronic health record, and therefore inaccessible. Second, treatment modalities will reflect available medical resources at a specific location (i.e., personnel, equipment, etc.), and will vary widely. This makes streamlined comparisons between treatment modalities (and medical facilities) impossible with this data set. A focused prospective clinical study at a single treatment facility would be better suited to support the reviewer’s critiques. We believe our data provide a basis to construct such a protocol that can delineate these additional knowledge gaps and variables that the reviewer mentions.

We have attempted to address the reviewer’s remaining concerns point-by-point:

1) The role of the biochemical profile in initial management.

In order to highlight the reviewer’s concerns pertaining to interpreting serum chemistries at initial management, we have amended the ‘Discussion – Limitations’ section with the following language:

[Line 288-292]

“While we have focused on the longitudinal nature of biochemical recovery, it is important to note that the initial clinical laboratory evaluation alone plays a vital role in early medical management of suspected EHS patients. For example, electrolyte abnormalities including hyponatremia will dictate intravenous administration of normal saline, and other treatment and medical decision-making.”

2) Influence of initial EHS treatment/management on the biochemical recovery profile.

The reviewer has provided several compelling case studies and case series to describe the influence of initial treatment/management on long-term biochemical recovery (Varghese et al., Sithinamsuwan et al., and Stearns et al.). We also value these reports, and they are now cited in the manuscript (see ref 39 & 40).

However, these reports narrowly reflect small patient cohorts (<30 patients per study), and the resources and practices at a small number of medical treatment facilities (<=2); we acknowledge the weakness of previous studies in the Discussion [Line 205-222].

Although this type of analysis is outside the scope of our study, the findings from these studies should not be undervalued, so we have added additional mention to the discussion section. See below:

[Line 293-299]

“Previous case reports and case series have shown that patient prognosis and biochemical recovery are largely influenced by initial treatment variables and modalities, most notable including the immediacy of aggressive cooling treatment (38,39). Our source data lacks the detail and resolution needed for this type of analysis. It is our hope that defining an archetypical biochemical recovery profile in a large cohort will facilitate comparison of other treatment modalities and practices between medical treatment facilities.”

3) All EHS events are not the same.

We appreciate the reviewer’s comment and acknowledge that there is a degree of variability in EHS patient presentation and the resulting biochemical profile. We have already presented the range, IQR, mean and median of each clinical lab value for each day, in order to appropriately describe the dispersion (ie. variability) of each analyte (See Supporting Tables in the Appendix). We added the language below to address the spectrum of EHS presentation:

Added [Line 260-263]

“However, based on the observed variability in biochemical recovery patterns among individuals, a 7-day post-incident physical examination including laboratory analysis would help identify less severe presentation of EHS, and facilitate an earlier return to activity.”

Finally, the authors discuss the cost of each EHS event as $5,337 per episode. There is no clear discussion that details how this is concluded; clearly this is markedly underestimated. I do not understand role in this manuscript.

Response to Reviewer Paragraph #2-2

We believe ‘EHS treatment cost’ is a vital summary statistic, and an important valuation that has not been previously described in the literature. This information pairs physiological cost on the patient with the financial cost on the health care system. We already addressed the ‘underestimation’ in the discussion section (see line 246-250). We also added a description of how this value was calculated:

Added [Line 79-83]

“This analysis included patient demographic variables including age, gender, body mass index, and race, and variables associated with patient care including clinical laboratory values, follow-up care information, and total treatment costs. Total treatment cost were calculated as the cumulative cost to the health care system including initial care and all follow-up or in-patient care associated with the initial injury.

Conclusion: this is an important contribution to the literature, however, it needs to be discussed in proper context.
